# A novel characteristic of a phytoplankton as a potential source of straight-chain alkanes

**DOI:** 10.1038/s41598-021-93204-w

**Published:** 2021-07-19

**Authors:** Naomi Harada, Yuu Hirose, Song Chihong, Hirofumi Kurita, Miyako Sato, Jonaotaro Onodera, Kazuyoshi Murata, Fumihiro Itoh

**Affiliations:** 1 Japan Agency for Marine-Earth Science and Technology, 2-15 Natsushima-cho, Yokosuka, Kanagawa 237-0061 Japan; 2 Toyohashi University of Technology, 1-1 Hibarigaoka, Tempaku, Toyohashi, Aichi 441-8580 Japan; 3 National Institute of Physiological Sciences, 38 Nishigonaka Myodaiji, Okazaki, Aichi 444-8585 Japan; 4 Phytopetrum Inc., 3A Tamaki House Bldg. 10-17, Akamichi, Uruma, Okinawa 904-2245 Japan

**Keywords:** Biological techniques, Biofuels

## Abstract

Biosynthesis of hydrocarbons is a promising approach for the production of alternative sources of energy because of the emerging need to reduce global consumption of fossil fuels. However, the suitability of biogenic hydrocarbons as fuels is limited because their range of the number of carbon atoms is small, and/or they contain unsaturated carbon bonds. Here, we report that a marine phytoplankton, *Dicrateria rotunda,* collected from the western Arctic Ocean, can synthesize a series of saturated hydrocarbons (*n*-alkanes) from C_10_H_22_ to C_38_H_78_, which are categorized as petrol (C_10_–C_15_), diesel oils (C_16_–C_20_), and fuel oils (C_21_–C_38_). The observation that these *n*-alkanes were also produced by ten other cultivated strains of *Dicrateria* collected from the Atlantic and Pacific oceans suggests that this capability is a common characteristic of *Dicrateria*. We also identified that the total contents of the *n*-alkanes in the Arctic *D. rotunda* strain increased under dark and nitrogen-deficient conditions. The unique characteristic of *D. rotunda* could contribute to the development of a new approach for the biosynthesis of *n*-alkanes.

## Introduction

Crude oil contains a series of alkanes that are thought to be produced mainly by thermal decomposition of organic compounds but are also produced by abiogenic formation in crustal fluids^1^ (e.g., polymerization of methane precursors^2^) and by bacterial biosynthesis^3^. Biosynthesis of hydrocarbons has been reported in bacteria^4–7^, phytoplankton^8–11^, and other organisms^12–14^. The biogenic hydrocarbons produced in all organisms are essential components or accidental products of biochemical processes. Phytoplankton are an important source of biogenic hydrocarbons, mainly C_15_ and C_17_ straight-chain alkanes and the C_21:6_ linear alkene^10,11,15^. Previous studies^10,11,15^ have reported that 24 species of phytoplankton have the capability to synthesize one or two major linear saturated or unsaturated hydrocarbons (C_15_, C_17_, or C_21:6_) along with trace amounts of other saturated hydrocarbons that have 12–25 carbon atoms. Hydrocarbons synthesized by these species tend to be unstable because the majority are unsaturated and hence vulnerable to attack and easily oxidized. Zooplankton and higher-trophic-level marine organisms also produce hydrocarbons, mainly pristane^16,17^. Marine bacteria produce mainly linear C_17_ and C_18_ alkanes^18^.

The stability and high diversity of natural marine hydrocarbons make them potentially important sources of fuels. Hydrocarbons used as energy sources are called “fossil hydrocarbons” because fossil hydrocarbons are generally formed or altered by reactions that involve decarbonylation of typically even-numbered fatty aldehydes in sediments or sedimentary rocks under high pressure and temperature over geologic time. However, oil composed mainly of linear alkanes and isoalkanes from C_11_ to C_26_ with an age estimated to be less than 1000 years^19,20^ has been discovered in the Uzon volcano caldera, Kamchatka. The chemical reduction of carbon dioxide by microorganisms has been suggested to be the likely mechanism of formation of this oil seep in the Uzon volcano caldera because the oil field is located 30–35 km from a hydrothermal spring^21^. However, the suggested mechanism is controversial because the responsible microorganisms have not been identified, and a series of chemical reactions involving microorganisms that would lead to the formation of a variety of linear alkanes with both even and odd numbers of carbons is still unknown, although new pathways for the biosynthesis of alkanes, mainly heptadecane and pentadecane, have been reported in cyanobacteria^3^.

In this study, we report that a marine phytoplankton, *Dicrateria rotunda* (*D. rotunda*) strain ARC1, collected from the western Arctic Ocean, has the novel capability to synthesize a series of linear, saturated hydrocarbons (*n*-alkanes) ranging from C_10_H_22_ to C_38_H_78_. This characteristic of *D. rotunda* is noteworthy because there has been no previous report of any organism with similar capability. We also demonstrate that the production of a series of *n*-alkanes is a robust characteristic of the *Dicrateria* genus, and we discuss the possible pathway of production and function of the *n*-alkanes. We characterize the detailed cellular structure of the ARC1 strain and estimate the environmental conditions that affect the production of *n*-alkanes. We also discuss the significance from the standpoint of biofuels of the capability of *D. rotunda* to produce *n*-alkanes vis-à-vis that of other, previously studied organisms.

## Results and discussion

### Characteristics of *n*-alkanes produced by *Dicrateria*

**Figure 1:**
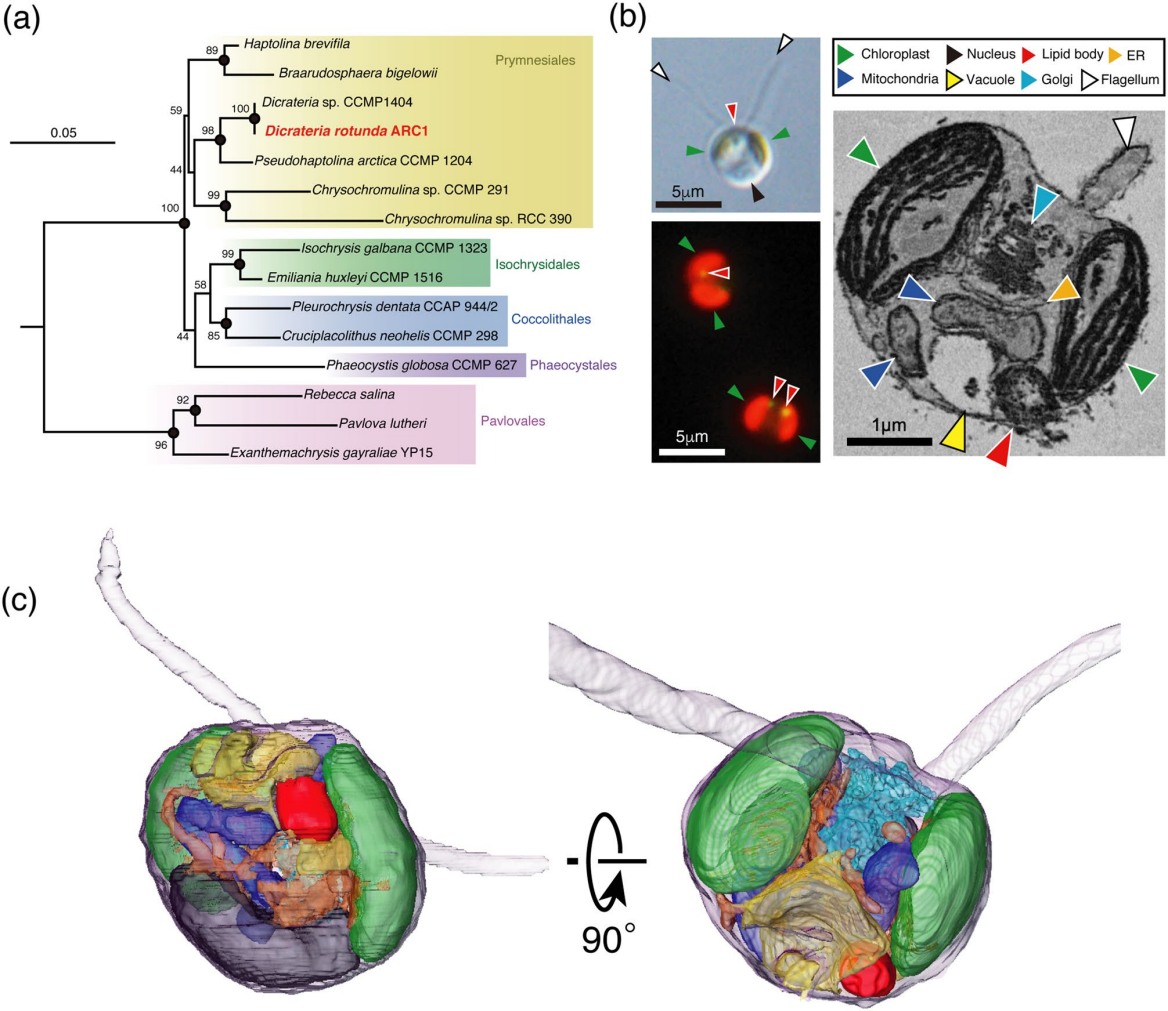
Phylogenetic and morphogenic characteristics of *Dicrateria rotunda *(*D. rotunda*) ARC1 strain. (**a**) Phylogenetic trees of 18S rRNA genes of the ARC1 strain and other selected haptophyte strains were estimated using the maximum likelihood method. Boot strap values of 1000 trials are shown. (**b**) Photographs of the ARC1 strain cultured with continuous light at 20 °C. Differential interference contrast microscopy (left upper), fluorescence microscopy with boron-dipyrromethene staining under blue excitation (left lower), and serial block-face scanning electron microscopy (right). Observed cellular structures are shown as colored arrows. (**c**) Detailed 3D structure of the cell of the ARC1 strain obtained by SBF-SEM. Each organelle has the same color as the corresponding arrow in (**b**).

A drastic reduction of sea ice has recently been observed in the western Arctic Ocean^22^. We have used the R/V *Mirai* of the Japan Agency for Marine-Earth Science and Technology^23–25^ to investigate the impacts of the ice reduction on lower-trophic-level organisms in the Arctic Ocean. During the cruise of August–October 2013, we isolated a phytoplankton strain from station 65 (70°00.06'N, 168°44.96'W, 10-m water depth). We found that the organism was a strain of *Dicrateria rotunda* (*D. rotunda*), class Haptophyceae, based on phylogenetic analysis of its 18S rRNA sequence (Fig. 1a)^26,27^. An axenic culture of the Arctic strain (ARC1) of *D. rotunda* was obtained via antibiotic treatment. The ARC1 strain can swim via two flagella, but it lacks coccoliths and organic lamella on its cell surface (Fig. 1b)*.* The presence of a chloroplast and a lipid body in which various kinds of hydrocarbons might accumulate^28^ was observed via optical microscopy and fluorescence microscopy with boron-dipyrromethene (BODIPY) fluorescence dye staining^29^ (Fig. 1b). Additional cellular components such as a nucleus, mitochondria, vacuole, endoplasmic reticulum (ER), and Golgi apparatus were observed by serial block-face scanning electron microscopy (SBF-SEM) (Fig. 1b,c, and Supplementary Video S1).

**Figure 2:**
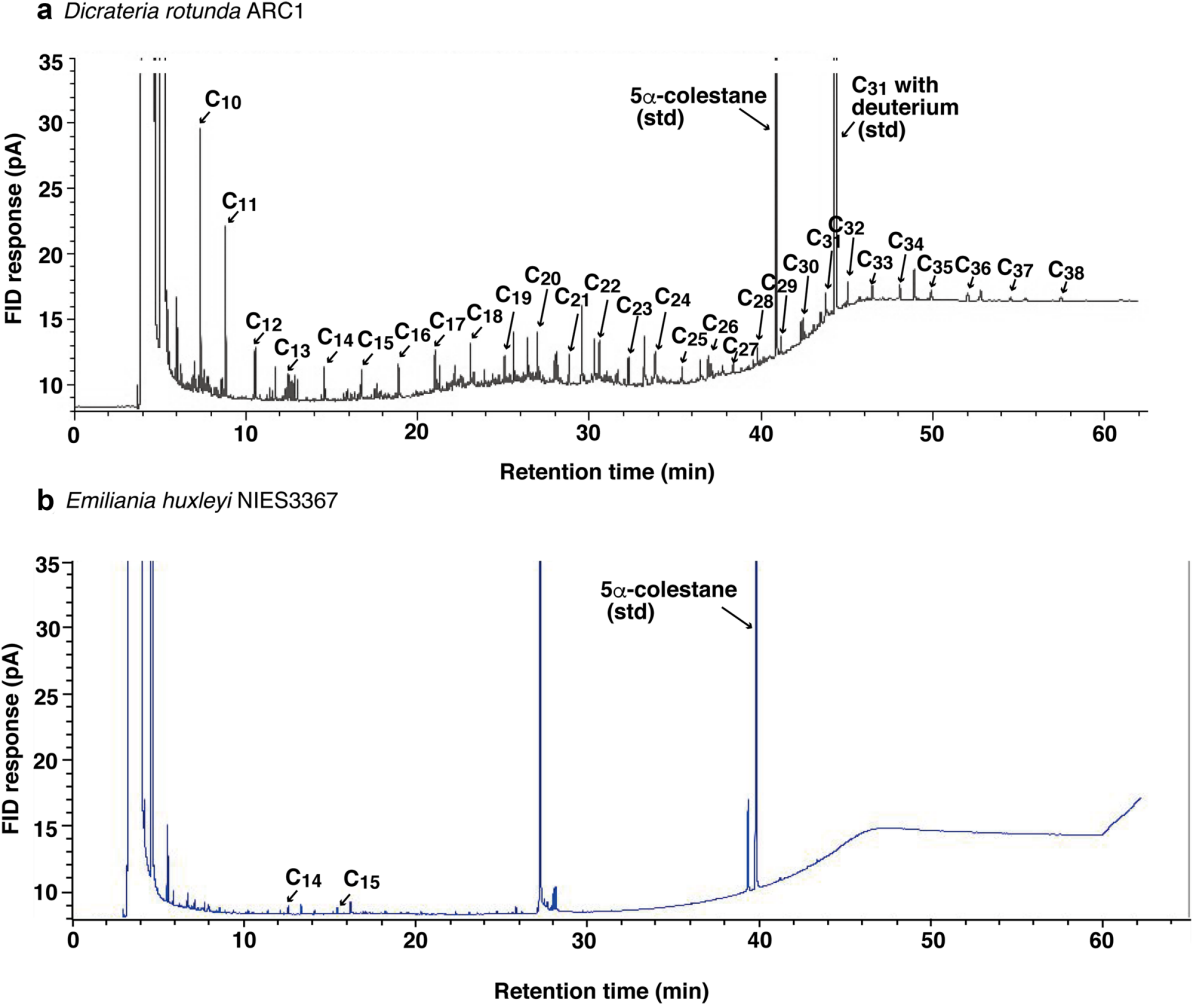
Gas chromatograms. (**a**) A series of alkanes from *D. rotunda* ARC1 strain and (**b**) *Emiliania huxleyi* NIES3367 strain*.* C_10_–C_38_ shows each *n*-alkane peak. The peak found at around 27 min is 5, 8, 11, 14, 17-Eicosapentanoic acid, methyl ester in the chromatogram of *Emiliania huxleyi* NIES3367 strain.

**Figure 3:**
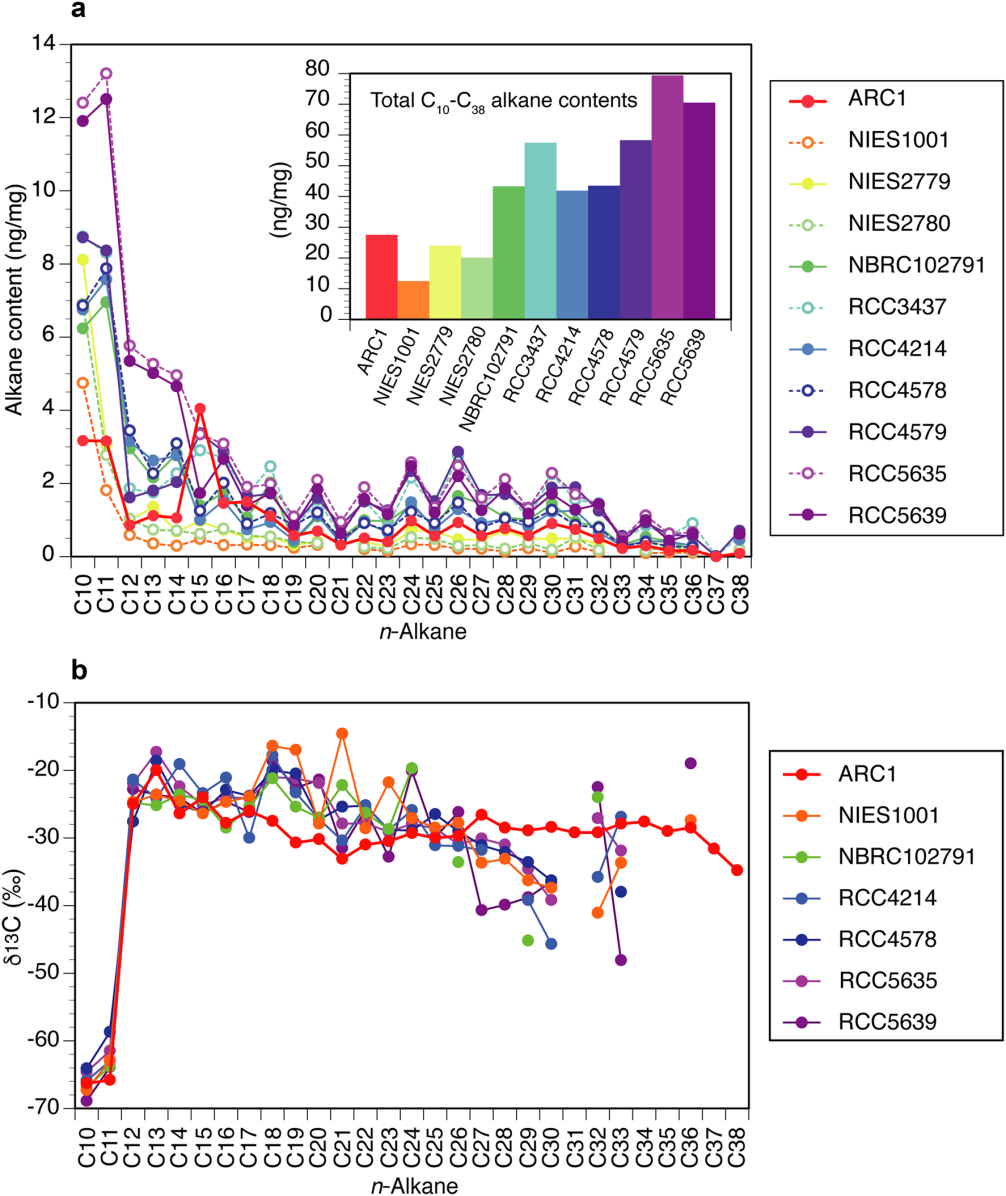
*n*-alkane concentrations and their compound-specific δ^13^C in the 11 *Dicrateria* strains. (**a**) Content of a series of *n*-alkanes, ng/mg cell dry weight and (**b)** their compound-specific δ^13^C values in the ARC1 strain and 10 other *Dicrateria* strains collected from ocean sites throughout the world and stored in culture collections. Total amounts of the *n*-alkanes of each strain are shown in the inset bar graph.

We analyzed the lipid contents of the ARC1 strain via gas chromatography-mass spectrometry (GC–MS) to identify every hydrocarbon based on the mass spectrum. The results of the gas chromatograph analysis revealed a consecutive series of straight-chain alkanes from C_10_ to C_38_ (C_10_H_22_–C_38_H_78_) in the neutral lipid fraction (Fig. 2a). A remarkable feature of the straight-chain alkanes produced by the ARC1 strain was that all straight-chain alkanes from C_10_ to C_38_ were present (Figs. 2a, 3a and Supplementary Table S1), and the composition of the alkanes was ideal for fuel oil. In terms of strains NIES1001, 2779, 2780 and NBRC10279, some alkanes were not detected (please see Supplementary Table S1). Another noteworthy characteristic was that the short-chain *n*-alkanes C_10_ (decane), C_11_ (undecane), and C_15_ (pentadecane) were the major components among all the *n*-alkanes, and there was no remarkable odd or even carbon number preference (Fig. 3a and Supplementary Table S1). The relatively high abundances of decane, undecane, and pentadecane are qualitatively consistent with the reported characteristics of the hydrocarbons produced by 24 species of algae, including 22 marine phytoplankton belonging to nine algal classes including Bacillariophyceae, Dinophyceae, Cryptophyceae, Haptophyceae, Euglenophyceae, Cyanophyceae, Rhodophyceae, Xanthophyceae, and Chlorophyceae^10,11,15,18,30–33^. The analyses of the alkanes produced by those 24 species^10,11,15,18,30,31^ have indicated that it is typical for the concentrations of one or two hydrocarbons (e.g., *n*-pentadecane in brown algae and *n*-heptadecane in red algae) to exceed the concentrations of the other alkanes by orders of magnitude^10^. Other consecutive *n*-alkanes from C_12_ to C_25_ have also been detected in species belonging to the Cyanophyceae, Rhodophyceae, Xanthophyceae, and Chlorophyceae^10,32^. However, there are no previous reports of any organisms having a capability like that of *D. rotunda* to synthesize a consecutive series of straight-chain *n*-alkanes from a short chain (C_10_) to a long chain (C_38_)*.* This capability of *D. rotunda* to synthesize *n*-alkanes might enable the production of fuel oil with an ideal composition.

*Dicrateria rotunda* is found not only in the Arctic Ocean (this study) but also in middle latitudes, according to information in culture collections throughout the world. We examined the capability to produce *n*-alkanes of four additional strains of *D. rotunda* (NIES1001, NIES2779, NIES2780, and NBRC102791) and six strains of *Dicrateria* sp. (RCC3437, RCC4217, RCC4577, RCC4578, RCC5635, and RCC5639). These additional strains were obtained from the culture collections of the National Institute for Environmental Studies (NIES), Japan, the NITE Biological Resource Center (NBRC), Japan, and the Roscoff Culture Collection (RCC), France. Notably, consecutive series of straight-chain alkanes from C_10_ to C_38_ were detected in all of the NIES, NBRC, and RCC strains (Fig. 3a and Supplementary Table S1). In contrast, almost no *n*-alkanes were detected except for small peaks of C_14_ and C_15_ in *Emiliania huxleyi* strain NIES3367 (Fig. 2b and Supplementary Table S1). *Emiliania huxleyi* is the most cosmopolitan of the species that belong to the algal class Haptophyceae and can live from the equator to subpolar latitudes under a variety of environmental conditions in the world oceans^34^. Thus, the capability to produce a consecutive series of straight-chain alkanes from short to long chains was a robust and unique characteristic of the genus *Dicrateria*. The content of total alkanes in the 11 strains of *Dicrateria* ranged from 12.5 to 79.4 ng/mg dry wt. (Fig. 3 and Supplementary Table S1), i.e., 0.01–0.08% of total cell dry weight. This range was lower than the range of 0.02–0.48% previously reported for 24 species of benthic algae belonging to the Chlorophyceae, Phaeophyceae, and Rhodophyceae^10^ as well as the range of 0.08–2.9% for six species of soil algae^18^.

*Botryococcus braunii* (*B. braunii*) is one of the most popular organisms used for the production of biodiesel because it has the ability to synthesize a large amount of oil per cell. The total hydrocarbon content of *B. braunii* varies dramatically, from 0.4% to 76% of cell dry weight, depending on the strain^35^. The hydrocarbons produced by *B. braunii* have three characteristics. First, in the odd-number *n*-alkenes from C_25_ to C_31_, there are two or three double bonds in the molecules. The biosynthesis of these compounds begins with oleic acid (C_18:1_, *n*-9). Two carbons are added at a time via acetyl-CoA, and odd-number *n*-alkanes are finally synthesized by decarbonylation. Second, the hydrocarbons include molecules with the stoichiometry C_n_H_2n-10_ (n = 30–37) that have a triterpene structure somewhere in the molecule. Third, the hydrocarbons include lycopadiene (C_40_H_70_)^35^. The hydrocarbons synthesized by not only *B. braunii* but also Botryococcane have been considered as potential sources of the long-chain *n*-alkanes in crude oil^36^. However, almost all the hydrocarbons produced by *B. braunii* are alkenes that are easily oxidized and are therefore not suitable for direct use as fuels. The capability of *Dicrateria* to produce a series of *n*-alkanes from C_10_ to C_38_ is superior to the hydrocarbon production capability of other known phytoplankton from the standpoint of the suitability of the hydrocarbons as fuels.

### Compound-specific carbon isotope ratios of the *n*-alkanes of *Dicrateria*

The compound-specific carbon isotope ratio (δ^13^C) of organic matter reflects the influence of environmental conditions on biological activity, the processes by which compounds are produced, and diagenesis over geological time scales. For example, the compound-specific carbon isotope ratio of organic matter has been used to identify the mechanisms by which *n*-alkanes are synthesized in petroleum source rocks or in oil shale during diagenesis^37,38^. Thus, compound-specific δ^13^C values might be expected to provide important information that could help elucidate the mechanisms by which *n*-alkanes are synthesized in a cell.

To obtain insight about how *n*-alkanes are synthesized in *Dicrateria*, we measured the compound-specific carbon isotope ratio of each alkane. The δ^13^C values of decane and undecane were relatively light and ranged from − 70 to − 60‰ (Fig. 3b and Supplementary Table S2). The δ^13^C of other alkanes ranged from − 35 to − 20‰ and were relatively heavy compared with those of decane and undecane (Fig. 3b and Supplementary Table S2). The δ^13^C composition of phytoplankton is controlled by various environmental parameters, but the immediate products of photosynthesis are generally depleted in ^13^C compared with the precursor inorganic carbon because of isotope fractionation^39^. Biosynthesis of a hydrocarbon is considered to involve elongation of a fatty acid followed by loss of the carboxyl carbon^40^. Fatty aldehydes can serve as the immediate precursors of *n*-alkanes because Co-porphyrin can catalyze the conversion of aldehydes to the corresponding alkanes with the loss of CO^41^. Based on the systematic depletion of ^13^C in products relative to precursors because of isotope fractionation during the biosynthesis of organic compounds^38^, the patterns of the compound-specific δ^13^C values of the *Dicrateria n*-alkanes implies that, the alkanes having heavier δ^13^C from − 35 to − 20‰ are the precursor of the biosynthesis of the decane and undecane having lighter δ^13^C from − 70 to − 60‰. Alternatively, decane and undecane may be synthesized through a biosynthesis pathway that is distinct from the biopathway of the other alkanes. In addition to analysis of the compound-specific δ^13^C values of the *n*-alkanes, we sought another approach—such as a physiological approach or discovery of the gene that controls production of *n*-alkanes—to verify our conclusions based on compound-specific isotope data about the mechanism involved in the biosynthesis of *n*-alkanes in the cell.

### Environmental conditions affecting *n*-alkane production in the ARC1 strain

**Figure 4:**
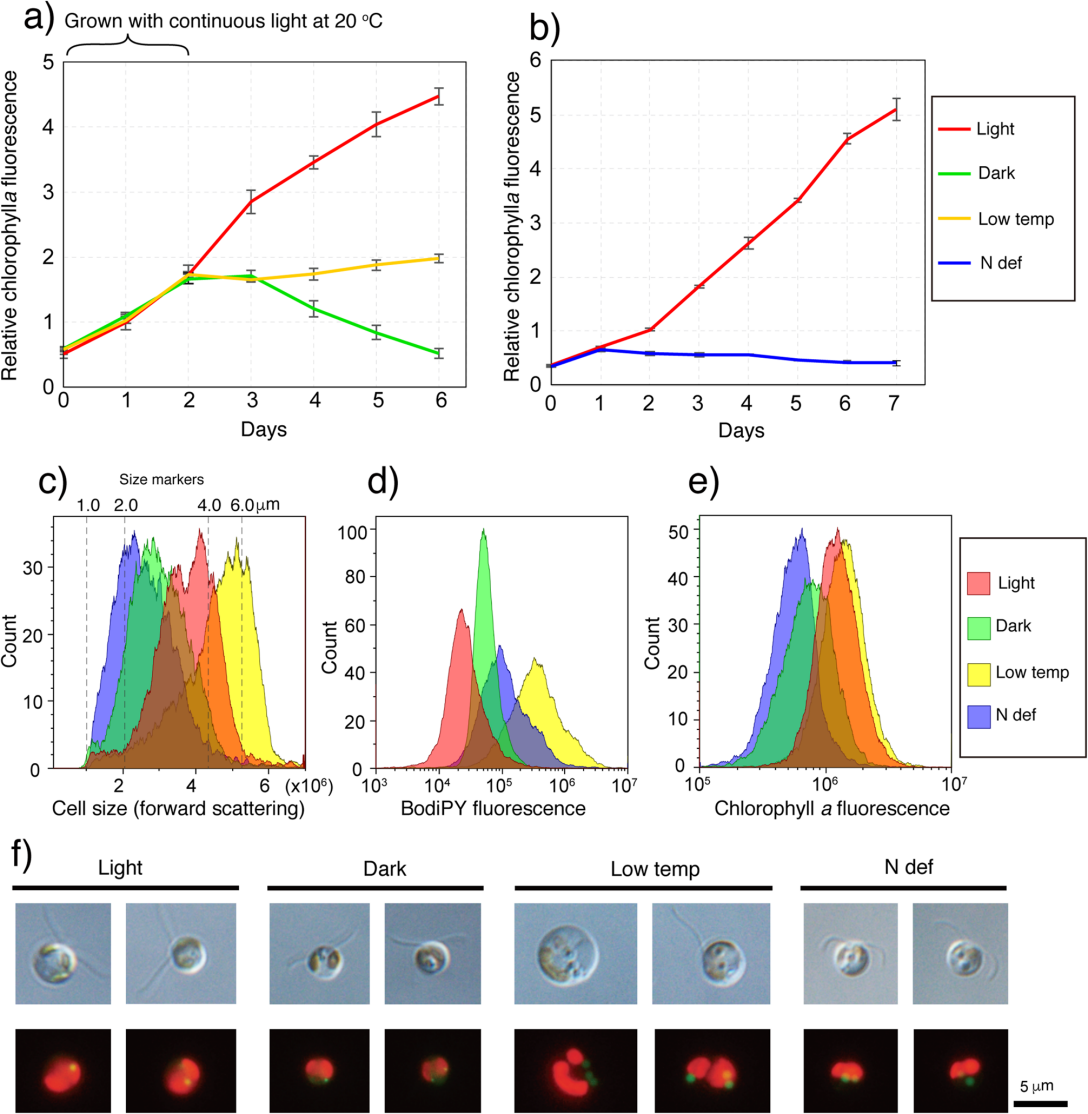
Effects of growth conditions on cell size and fluorescence from BODIPY and Chl.*a*. (**a**,**b**) Fluorescence from Chl.*a* was monitored in the ARC1 strain grown under four different culture conditions: continuous light at 20 °C (red); continuous dark at 20 °C (green); continuous light at 4 °C (yellow); and continuous light under nitrogen-deficient conditions at 20 °C (blue). Error bars indicate standard deviations of triplicate experiments. (**c–e)** Flow cytometer analysis of cell size (**c**), and intensity of BODIPY (**d**) and Chl.*a* (**e**) fluorescence of the ARC1 strain. (**f)** Light (upper) and fluorescence (lower) microscopic observations of the ARC1 strain under four different growth conditions. Red and green fluorescence from Chl.*a* and BODIPY, respectively were detected under blue light excitation.

Changes in the environmental conditions have been reported to promote lipid accumulation in diverse algal strains^42^. We examined the effects of several key parameters, including light, temperature, and inorganic nitrogen, on *n*-alkane production in the ARC1 strain. The ARC1 strain was grown under continuous light at 20 °C for two days and then grown for four days under three different conditions of continuous light at 20 °C (light), continuous dark at 20 °C (dark), or continuous light at 4 °C (low temperature) (Fig. 4a). In another experiment, the ARC1 strain was grown in a nitrogen-depleted medium with continuous light at 20 °C for seven days to expose the cells to nitrogen-deficient condition (Fig. 4b). Flow cytometer analysis showed two peaks in the distribution of cell size grown under light condition, which is probably attributed to the two different phases in the cell cycle of the ARC1 strain (Fig. 4c). This analysis revealed decreases in cell size and chlorophyll *a* (Chl.*a*) fluorescence and an increase of BODIPY fluorescence under both dark and nitrogen-deficient conditions (Fig. 4c,e). In contrast, increases in cell size and BODIPY fluorescence were observed in the cells under low-temperature conditions, but there was little change of Chl.*a* fluorescence (Fig. 4c–e). SBF-SEM analysis showed that cells grown under low-temperature conditions formed a more distinct lipid body, and there were increases in the number and total volume of the lipid bodies (Supplementary Fig. S1 and Supplementary Video S2).

**Figure 5:**
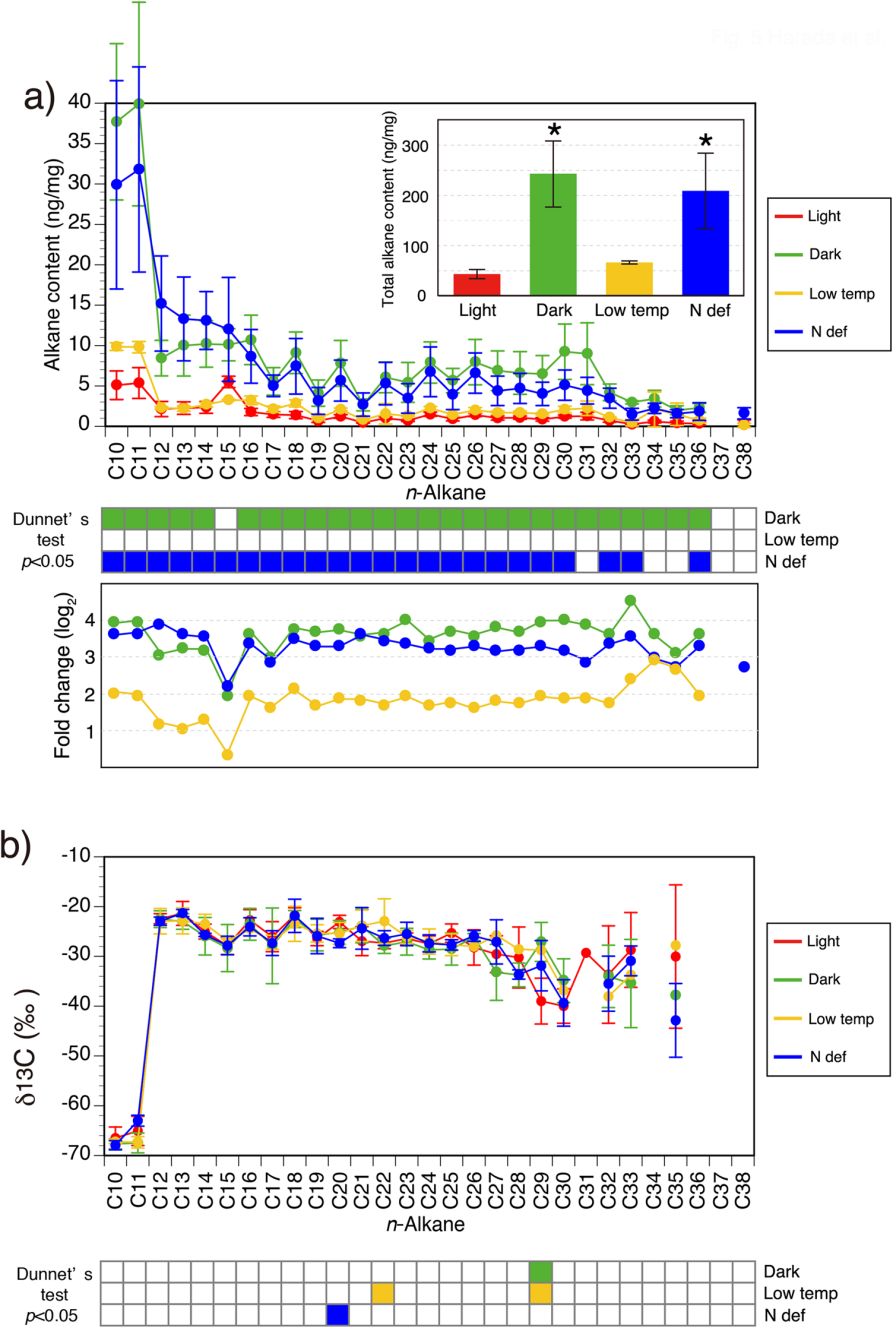
Effect of environmental conditions on the concentrations and compound-specific δ^13^C of *n*-alkanes in the ARC1 strain. (**a**) Concentration of a series of *n*-alkanes per dry weight; (**b)** *n*-alkane compound-specific δ^13^C composition. Both were quantified in the ARC1 strain grown under four different culture conditions: continuous light at 20 °C (light); continuous dark at 20 °C (dark); continuous light at 4 °C (low temp); and continuous light under nitrogen-deficient conditions at 20 °C (N def). Error bars indicate standard deviations (*n* = 3 for dark, low temp, and N def conditions; *n* = 6 for light condition). Significant differences in the total amount of the series of *n*-alkane concentrations between light and low temp, dark, or N def conditions were detected by Dunnett’s test (asterisks show *p* < 0.05 in the inset figure). For each alkane compound, environmental conditions showing the significant difference in the Dunnett’s test (*p* < 0.05) are shown as filled boxes. Lines, bars, and boxes are colored to reflect growth conditions.

However, GC analysis showed that the behavior of the *n*-alkane contents differed from that of the lipid bodies. Total content of *n*-alkanes per dry cell weight was 45.1 ng/mg for light condition, 242 ng/mg for dark condition, 65.9 ng/mg for low-temperature condition, and 209 ng/mg for nitrogen-deficient condition (Supplementary Table S1). The range of the total *n*-alkane content of the ARC1 strain per dry cell weight under the four different conditions (0.005–0.024%) was lower than those of 0.02–2.9% previously reported for benthic and soil algae^10,18^. The total content of *n*-alkanes increased 5.6-fold (Dunnett’s test; *p* < 0.05) under dark condition and 4.8-fold (Dunnett’s test; *p* < 0.05) under nitrogen-deficient condition compared with continuous-light conditions (Fig. 5a inset and Supplementary Table S1). The increases in most of the carbon numbers of *n*-alkanes were significant (Dunnett’s test; *p* < 0.05) (Fig. 5a, filled boxes). The total *n*-alkane content was 1.5-fold larger under low-temperature conditions than under continuous-light conditions, but the difference was not significant (Fig. 5a, inset). These results demonstrate that the *n*-alkanes of the ARC1 strain accumulated under dark and nitrogen-deficient conditions, but they did not accumulate to a significant extent under low-temperature condition, even though the numbers and volumes of the lipid bodies increased. The fact that we did not observe a substantial treatment effect on the fold change of the *n*-alkanes versus the treatment effect on their carbon numbers (Fig. 5a) implies that the composition of the *n*-alkanes was not affected. The dark-induced accumulation of the alkanes in the ARC1 strain implies that the alkanes did not serve as an energy source in the absence of photosynthesis. The increase of the *n*-alkane content under nitrogen-deficient condition, during which the growth of the cells was suppressed, suggests that *n*-alkanes were produced by a growth-independent mechanism. We also measured the δ^13^C of the *n*-alkanes of the ARC1 strain under the four different culture conditions, but the fact that most of the δ^13^C values did not change significantly (Fig. 5b and Supplementary Table S2) implies that the pathway of biosynthesis of the *n*-alkanes was not changed by the different growth conditions.

Some strains of the order Isochrysidales of Haptophyte algae, including *Emiliania huxleyi*, (Fig. 1a) produce unique long-chain unsaturated methyl- or ethyl-ketones called alkenones^43^. The alkenones are thought to serve as metabolic storage molecules^44^. The capability to produce alkenones has not been observed in other genera of the order Pryminesiales^45^. *Chrysochromulina tobin*, which belongs to the order Pryminesiales of the phylum Haptophyte (Fig. 1a), contains high concentrations of saturated and unsaturated fatty acids ranging from C_14_ to C_22_ in the lipid bodies, and it uses those fatty acids for energy storage during a light/dark cycle^46^. These observations suggest that substantial diversity exists in the kinds of hydrocarbons produced by each genus of Haptophyte. Culture experiments have found that some cyanobacteria synthesize C_15_–C_19_ saturated hydrocarbons and accumulate them in their membranes^47^. In an experiment using cyanobacteria that were deficient in *n*-alkanes, the sizes of the cells increased because the membranes were unstable^47^. It has been suggested that *n*-alkanes are needed in membranes to reduce membrane permeability, to control cell size, to enable the cell to grow, and to give the cell membranes the flexibility needed for optimal cell division^47^. Lea-Smith et al.^47^ have also suggested that it is essential that hydrocarbons accumulate in a cell membrane and that such accumulation enables microalgae to minimize risks and to grow rapidly under optimal growth conditions. One possible function of the *n*-alkanes in *Dicrateria* might be keeping the membrane system in optimal condition during the shrinking of cell volumes under the stress of dark and nitrogen-deficient growth conditions (Fig. 4c). Further analysis is required to elucidate the physiological roles and the location of the *n*-alkanes in a *Dicrateria* cell.

## Materials and methods

### Samples

Surface water was collected at St.65 (70° 0.06′ N, 168° 44.96′ W) in the Chukchi Sea, western Arctic Ocean, during cruise MR13-06 of the R/V *Mirai*. Basic feature of surface water sample was as follows; water temperature: 2.56 °C, salinity: 31.4, oxygen concentration: 334.7 µmol/kg, nitrate concentration: 0.05 µmol/kg, nitrite concentration: 0.02 µmol/kg, silicate concentration: 0.41 µmol/kg, phosphate concentration: 0.395 µmol/kg. An aliquot of seawater was separated into two batch culture bottles containing natural seawater enriched with nutrients as specified for 1/10 MNK medium^48^. Cultures were incubated at 4 °C on a 16-h light:8-h dark cycle of illumination on board the ship. An aliquot (20 mL) of the seawater cultured on board the ship was transferred to MA-ESM medium^49^, and the cells were further cultured at 5 °C and 10 °C under a photoperiod illumination of 20 µmol quanta m^−2^ s^−1^ on a 16-h:8-h L:D cycle. We succeeded in isolating an axenic Arctic strain (ARC1) by treatment with the antibiotics G-418 (200 mg/mL), kanamycin (20 mg/mL), spectinomycin (20 mg/mL), and ampicillin (100 mg/mL) and by single-cell isolation under a microscope. The axenicity was checked by microscopic observation of the cells of the ARC1 strain that had been cultured on a Marine Broth 2216 agar plate (BD Bioscience) for 1 week at 25 °C. The axenic culture of the *D. rotunda* ARC1 was deposited in the culture collection of the National Institute of Technology and Evaluation (NITE), Japan with accession number FERM BP-22332. We also obtained other *Dicrateria* strains from culture collections. Those strains included *D. rotunda* from the National Institute for Environmental Studies (NIES), Japan (http://mcc.nies.go.jp/index_en.html) and Roscoff Culture Collection (RCC), France (http://www.roscoff-culture-collection.org/). The strains were *D. rotunda* Reynolds emend. J. C. Green & Pienaar (NIES1001, NIES2779, NIES2780, and NBRC102791 collected from the North Pacific Ocean) and *Dicrateria* sp. (RCC3437, RCC4577, and RCC4578 collected from the South Atlantic Ocean; RCC5635 and RCC5639 collected from the South Pacific Ocean; and RCC4214 collected from North Atlantic Ocean). The ARC1 strain was maintained in f/2 medium^50^ under aeration, aseptic conditions, 20 °C, and 10 µmol quanta m^−2^ s^−1^ of continuous illumination. The other strains were grown under the same light and temperature conditions in K^51^, K/2^51^, or ESM^52^ liquid media as described in Supplementary Table S1. The culture in stationary phase was used for measurements of the concentration of *n*-alkanes and their compound-specific stable carbon isotope ratios.

### Phylogenetic analysis

The 18S rRNA sequence of *D. rotunda* (GenBank accession LC519889) was obtained from a draft genome assembly using the Pacbio RSII sequencer (Hirose et al., in preparation). Representative haptophytes and their 18S rRNA sequences are listed in Supplementary Table S1 as follows: *Pavlova lutheri* (AF102369), *Rebecca salina* DHmm3W3 (KU561125), *Exanthemachrysis gayraliae* YP15 (AF106060), *Phaeocystis globose* CCMP 627 (AJ278035), *Isochrysis galbana* CCMP1323 (HM149540), *Emiliania huxleyi* CCMP 1516 (AHAL01000301), *Cruciplacolithus neohelis* CCMP 298 (AJ246262), *Pleurochrysis dentata* CCAP 944/2 (KJ756811), *Imantonia* sp. CCMP1404 (AM491015), *Pseudohaptolina arctica* CCMP1204 (AM491016), *Braarudosphaera bigelowii* Funahama-T3 (AB478412), *Haptolina brevifila* Kawachi (AM490995), *Chrysochromulina* sp. CCMP291 (JWZX01002122), and *Chrysochromulina* sp. RCC 390 (KT878670). In addition, *Guillardia theta* CCMP2712 (AEIE01003291), a member of the Cryptophyceae, was used as an outgroup. These sequences were aligned with MAFFT version 7 online with default parameters^53^. The phylogenetic tree was estimated by the maximum likelihood method using RAxML (version 8.2.11)^54^. The GTRGAMMA^54^ model was used with 1000 bootstrap iterations (options -f a -m GTRGAMMA -# 1000).

### Scanning electron microscopy

For serial block-face scanning electron microscopy (SBF-SEM), cells were grown at 20 °C or 4 °C in a 400 mL flask, fixed with 3% glutaraldehyde by volume for 30 min, and collected by centrifugation at 3000 × g at room temperature for 5 min. The cells were washed with phosphate-buffered saline solution (PBS, pH 7.5) to remove glutaraldehyde and then post-fixed with 2% osmium tetroxide and 1.5% potassium ferrocyanide in PBS for one hour. The cells were washed with distilled water and incubated in 1% thiocarbohydrazide solution for 20 min. They were then washed with distilled water and fixed again with 2% osmium tetroxide in distilled water at room temperature for 30 min. After washing with distilled water, the fixed cells were stained *en bloc* with 1% uranyl acetate in distilled water at 4 °C overnight and with 0.2 mol/L lead aspartate (pH 5.5) at 60 °C for 30 min. The cells were washed with distilled water and dehydrated with a series of ethanol washes (50–100%) at 4 °C and dehydrated with acetone at room temperature. Finally, they were infiltrated with Durcupan (Sigma-Aldrich, St Louis, Missouri, USA) resin and polymerized at 60 °C for three days. The resin block containing the sample was trimmed and glued onto an aluminum rivet with a conductive epoxy resin (SPI Conductive Silver Epoxy; SPI Supplies and Structure Probe, Inc., West Chester, PA, USA) and then coated with gold using an ion coater. A serial block-face scanning electron microscope (MERLIN; Carl Zeiss Microscopy, Jena, Germany) equipped with a OnPoint back-scattered electron detector (Gatan Inc., Pleasanton, CA, USA) was used to slice and image the specimens. The scanning electron microscope was operated at a low accelerating voltage of 1.2 kV. The serial images were automatically acquired by using Gatan Digital Micrograph software. All images were taken at an image size of 8192 × 8192 pixels (pixel size = 4 nm) with a thickness of 50 nm. The image series was automatically aligned using Fiji (http://fiji.sc/Fiji) and cropped around each single cell. Segmentation of the volume data was performed with Amira version 5.4.5 (Thermo Fisher Scientific, Waltham, MA, USA).

### Effect of growth environment and flow cytometric characterization

To investigate the effects of growth conditions on *n*-alkane production, 40 mL of the culture of the ARC1 strain of *D. rotunda* at stationary phase was transferred to 400 mL of fresh f/2 medium and cultured in 1 L Erlenmeyer flasks containing 400 mL of f/2 medium. The ARC1 strain was cultured under continuous light (flasks were aerated; the culture aseptic; and continuous light was supplied by a white fluorescent lamp at an intensity of 10 µmol quanta m^−2^ s^−1^) at 20 °C. *Dicrateria rotunda* was cultured under these conditions for two days, and the ARC1 strain was then transferred and grown under four different conditions for four days: (1) continuous light at 20 °C; (2) continuous darkness at 20 °C; (3) continuous light at 4 °C and; (4) continuous light with nitrogen deficiency at 20 °C. Because the optimum cell growth of the ARC1 strain was observed at a low cell density, the growth of the cells was estimated by the measurement of red fluorescence of Chl.*a* using a Qubit 4 fluorometer (Thermo Fisher Scientific) once each day instead of by measurement of optical density using a spectrophotometer. Each of the four different culture experiments was run in triplicate (total of 12 flasks). After cultivation for a total of six days, cells were collected by centrifugation for 10 min at 3780×*g* and frozen at −80 °C for gas chromatography-mass spectrometry and gas chromatography analyses of the *n*-alkane compositions and their contents, respectively. About 30 mL of culture was centrifuged for 5 min at 3000×*g*, resuspended in 500 µL of f/2 medium, and subjected to flow cytometry and microscopy. The fluorescence intensity of Chl.*a*, BODIPY, and cell size were measured as R-fluorescence, G-fluorescence, and forward-scattering light, respectively, via 488-nm blue laser excitation using a flow cytometer (CytoFLEX, Beckman Coulter, Inc., Indianapolis, IN, USA). A Flow Cytometry Size Calibration Kit (Invitrogen) was used as the standard maker for the forward-scattering analysis. The cells of the NIES and RCC strains were prepared for *n*-alkane quantification as described for the ARC1 strain under continuous illumination at 20 °C. For differential interference contrast microscopy and fluorescence microscopy, a 1/100 volume dilution of BODIPY 505/515 solution (Thermo Fischer Scientific, 10 µg/mL in DMSO) was added to the cell solution and observed using an IX-73 microscope (Olympus Corporation, Japan) equipped with a DP-80 camera (Olympus Corporation, Japan). Green BODIPY fluorescence and red Chl.*a* fluorescence were observed with blue light excitation (460–495 nm) using a U-FBW mirror unit (Olympus Corporation, Japan).

### Alkane analysis

A sample of 20–30 mg of dry cells of *D. rotunda* was placed in a stainless-steel cell, and all lipids were extracted with a mixed solvent of dichloromethane and methanol (9:1 by volume) using an accelerated solvent extractor (ASE-200, DIONEX Japan Ltd., Japan) under 100 °C and 1500 psi (= 10,343 kPa) for 20 min. The solvent mixture of dichloromethane and methanol was concentrated to a volume of less than 1 mL with nitrogen gas. Ten milliliters of a mixture of 0.5 mol/L potassium hydroxide in methanol was added to the concentrated solvent mixture of dichloromethane and methanol to saponify the mixture at 80 °C for 2 h. After saponification, an aliquot of hexane was added to the cooled, saponified solution, and the solution was shaken to dissolve the lipid fraction into the hexane layer. The hexane layer was taken up and transferred into a glass tube. The hexane fraction was concentrated under nitrogen gas to an approximate volume of 1 mL. This concentrated hexane fraction was transferred to a silica gel column, and the *n*-alkane fraction was extracted with hexane by silica gel column chromatography (Rapid Trace SPE Workstation, Biotage Japan Ltd., Tokyo, Japan). The series of *n*-alkanes was identified with a gas chromatograph-mass spectrometer (Agilent 7200GC/Q-TOF mass spectrometer, Agilent Technologies Japan Ltd., Tokyo, Japan) equipped with a glass column (DB-5 ms: 30 m long, 0.25 mm inner size, 0.25 µm thickness). A series of *n*-alkanes was also analyzed with a gas chromatograph (Agilent 6890 N, Agilent Technologies Japan Ltd., Tokyo, Japan) equipped with a glass column (HP-5MS, Agilent: 60 m long, 0.32 mm inner-diameter, and 0.25 µm thickness) and flame-ionization detector (FID). The oven program started at 60 °C, increased at 10 °C/min up to 120 °C, 5 °C/min up to 310 °C, and was then held constant for 22 min at 310 °C. The drifting baseline was subtracted from the peak area when each FID detector response (peak area) was calculated. A series of *n*-alkanes was quantified via calibration curves estimated from the relationship between the concentration of each C_10_–C_38_ alkane standard material (GL Science Inc., Tokyo, Japan) and the FID detector response. The yield of C_10_–C_38_ alkanes over all steps from extraction of lipids to the GC measurement was 60–70% for C_10_–C_14_ alkanes and more than 95% for alkanes larger than C_15_. The yield of short-chain *n*-alkanes was lower than that of long-chain alkanes because of the high volatility of the former. Precision of replicate measurements of *n*-alkane concentrations was within 3% for C_10_–C_38_ alkane concentrations (*n* ≥ 3). Dunnett’s test was performed using multicomp ver.1.4.14. of the R package (version 4.0.3, 2020, https://www.R-project.org).

### Compound-specific stable carbon isotope analysis of alkanes

Compound-specific stable carbon isotope analysis of *n*-alkanes was carried out with a gas chromatograph/combustion/isotope ratio mass spectrometer (GC/C/IRMS, DeltaPlus XP, Thermo Fisher Scientific, K.K., Tokyo, Japan) equipped with a glass column (HP-1MS, Agilent: 60 m long, 0.32 mm inner-diameter, 0.25 µm thickness). The oven program of the GC started at 60 °C for 1 min, increased at 2.5 °C/min up to 80 °C, 40 °C/min up to 120 °C, 5 °C/min up to 310 °C, and was then held for 20 min at 310 °C. The temperature of combustion was 950 °C. Precision for replicate measurements of individual compounds in standard C_10_–C_38_ hydrocarbons (GL Science Inc., Tokyo, Japan) using this configuration ranged between 0.2‰ and 0.5‰ for *n*-alkanes. Prior to the carbon isotope analysis, the CO_2_ reference gas was calibrated relative to Pee Dee Belemnite (PDB) using the primary NBS 19 and 22 standards. Instrument performance was routinely checked using the previously prepared *n*-alkane standard solutions of *n*-eicosane (GL Science Inc., Tokyo, Japan; its *δ*^13^C was determined to be − 31.9‰ by SI Science Ltd., Japan) and *n*-pentatriacontane (GL Science Inc., Tokyo, Japan; its *δ*^13^C was determined to be − 30.7‰ by SHOKO Science CO. Ltd., Kanagawa, Japan). Isotopic compositions of *n*-alkanes were reported relative to PDB, and *δ*^13^C values were averaged over replicate measurements (*n* ≥ 3).

## Supplementary Information

Supplementary Information.

Supplementary Video 1.

Supplementary Video 2.
