# Supplementary material for: A novel characteristic of a phytoplankton as a potential source of straight-chain alkanes
Source: Sci Rep. 2021 Jul 19;11:14190. doi: 10.1038/s41598-021-93204-w (PMC8286971; doi:10.1038/s41598-021-93204-w)
Supplement: Supplementary file 1 — Supplementary Information. [file 41598_2021_93204_MOESM1_ESM.pdf]

A novel characteristic of a phytoplankton as a potential source of straight-chain alkanes

Naomi Harada[1*†], Yuu Hirose[2†], Song Chihong[3], Hirofumi Kurita[2], Miyako Sato[1], Jonaotaro Onodera[1], Kazuyoshi Murata[3] & Fumihiro Itoh[4]

[1]Japan Agency for Marine-Earth Science and Technology, 2-15 Natsushima-cho, Yokosuka, Kanagawa, 237-0061, Japan. [2]Toyohashi University of Technology, 1-1 Hibarigaoka, Tempaku, Toyohashi, Aichi, 441-8580, Japan. [3]National Institute of Physiological Sciences, 38 Nishigonaka Myodaiji, Okazaki, Aichi, 444-8585, Japan. [4]Phytopetrum Inc., 3A Tamaki House Bldg. 10-17, Akamichi, Uruma, Okinawa, 904-2245, Japan.
[†]These authors contributed equally to this work.

Supplementary Figure S1. Quantification of the number and volumes of lipid bodies in the SBF-SEM analysis of the ARC1 strain. (**a**) Cell diameter, (**b**) number and (**c**) total volume of lipid bodies per cell were quantified in the SBS-SEM analysis. For each analysis, 50 cells grown at 20 °C (black bar), and 50 cells grown at 4 °C (gray bar) were quantified (total, 100 cells).

Supplementary Table S1. *n*-alkane concentrations of the ARC1 strain and ten cultivated strains of *Dicrateria* collected from the Atlantic and Pacific oceans.

Supplementary Table S2. Compound-specific $\delta^{13}C$ of *n*-alkanes of the ARC1 strain and ten cultivated strains of *Dicrateria* collected from the Atlantic and Pacific oceans, and the ARC1 strain under four different culture conditions.

Supplementary Movie S1. SBS-SEM images and construction of 3D structure of the ARC1 strain grown under light condition at 20 °C. Chloroplast (green), nucleus (black), lipid bodies (red), endoplasmic reticulum (ER, orange), mitochondria (blue), vacuole (yellow), Golgi apparatus (cyan) are colored as indicated.

Supplementary Movie S2. SBS-SEM images and construction of 3D structure of the ARC1 strain grown under light condition at 4 °C. Chloroplast (green), nucleus (black), lipid bodies (red), endoplasmic reticulum (ER, orange), mitochondria (blue), vacuole (yellow), Golgi apparatus (cyan) are colored as indicated.

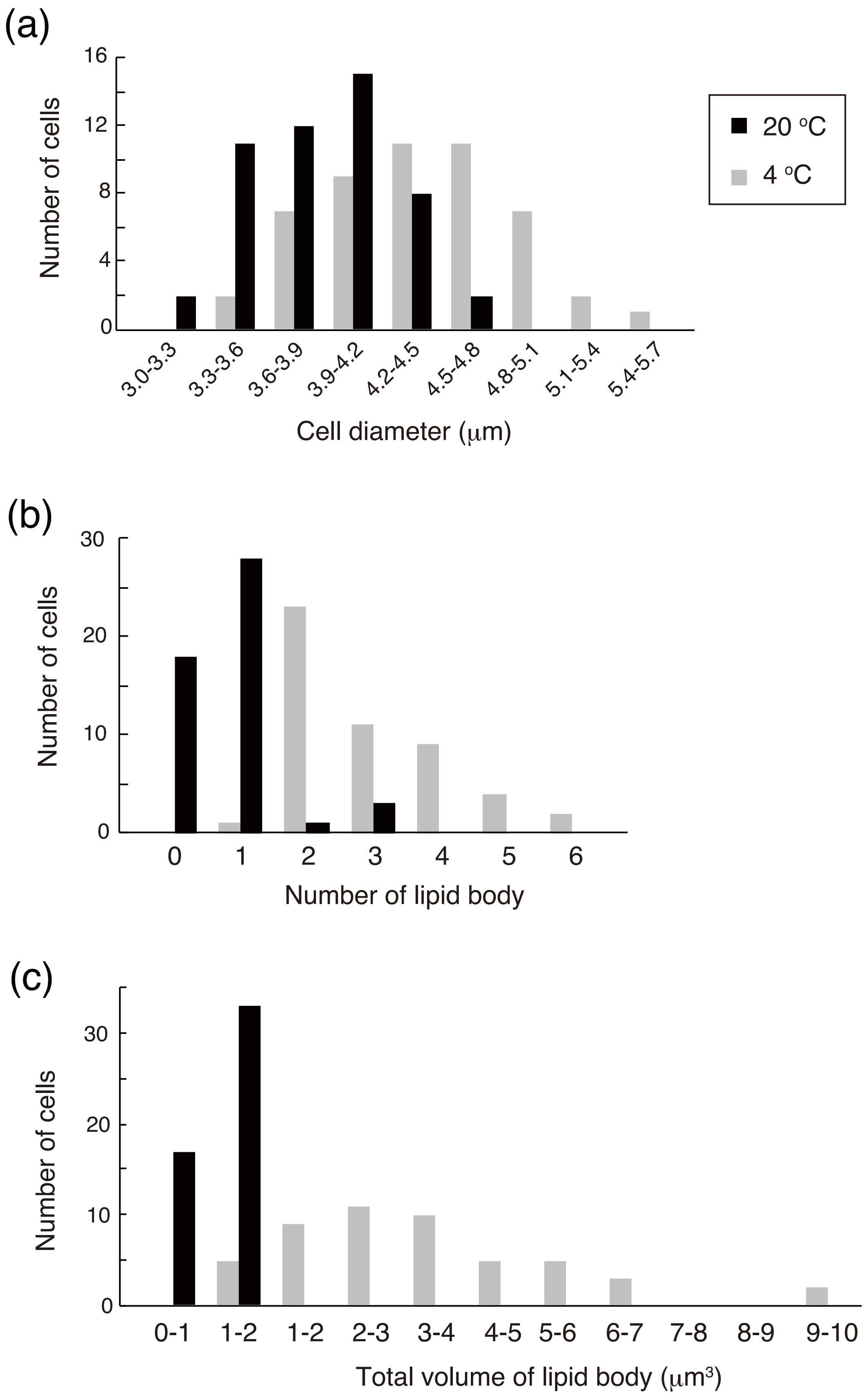
(a)
16
12
8
4
0
Number of cells
20 °C
4 °C
3.0-3.3
3.3-3.6
3.6-3.9
3.9-4.2
4.2-4.5
4.5-4.8
4.8-5.1
5.1-5.4
5.4-5.7
Cell diameter (μm)
(b)
30
20
10
0
Number of cells
0
1
2
3
4
5
6
Number of lipid body
(c)
30
20
10
0
Number of cells
0-1
1-2
1-2
2-3
3-4
4-5
5-6
6-7
7-8
8-9
9-10
Total volume of lipid body (μm3)

Supplementary Table S1 The concentration of linear alkane (ng/mg dried cells) in the Dicrateria strains grown with different liquid media or different conditions.

Strain	Arctic strain*	NIES1001*	NIES2779*	NIES2780*	NBRC102791*	RCC3437*	RCC4214*	RCC4578*	RCC4579*	RCC5635*	RCC5639*	Arctic strain		Arctic strain		Arctic strain		Arctic strain		*Emiliania huxleyi* (NIES-3367)
Growth medium	f/2	ESM	ESM	ESM	K	K/2	K/2	K/2	K/2	K	K	f/2		f/2		f/2		f/2		f/2
Growth condition	Continuous light at 20 °C	Continuous light at 20 °C	Continuous light at 20 °C	Continuous light at 20 °C	Continuous light at 20 °C	Continuous light at 20 °C	Continuous light at 20 °C	Continuous light at 20 °C	Continuous light at 20 °C	Continuous light at 20 °C	Continuous light at 20 °C	light (24 h) 20 °C		dark (24 h) 20 °C		light (24 h) and low temperature (4 °C)		light (24 h) with nitrogen deficiency		Continuous light at 20 °C
Alkane content	ng/mg	ng/mg	ng/mg	ng/mg	ng/mg	ng/mg	ng/mg	ng/mg	ng/mg	ng/mg	ng/mg	ng/mg	deviation (*n* = 6)	ng/mg	deviation (*n* = 3)	ng/mg	deviation (*n* = 3)	ng/mg	deviation (*n* = 3)	ng/mg (*n* = 2)
C10	3.17	4.74	8.11	6.9	6.23	8.74	6.75	6.86	8.72	12.4	11.9	5.09	1.77	37.7	9.68	9.87	0.474	29.9	12.9	0.000
C11	3.16	1.81	3.05	2.77	6.95	8.30	7.57	7.87	8.36	13.2	12.5	5.36	1.91	39.9	12.6	9.8	0.708	31.8	12.7	0.000
C12	0.864	0.586	0.989	1.03	2.95	1.86	3.15	3.44	1.61	5.76	5.34	2.14	0.934	8.44	2.20	2.28	0.147	15.2	5.89	0.000
C13	1.12	0.353	1.355	0.731	2.16	1.75	2.62	2.27	1.80	5.26	5.01	2.26	0.778	10.0	3.77	2.20	0.325	13.3	5.19	0.000
C14	1.06	0.291	0.677	0.708	2.81	2.27	2.76	3.09	2.03	4.95	4.65	2.34	0.729	10.2	2.88	2.68	0.283	13.1	3.58	0.001
C15	4.05	0.477	0.972	0.616	1.39	2.90	0.999	1.25	3.37	3.34	1.73	5.50	0.706	10.1	1.99	3.26	0.086	12.0	6.43	0.001
C16	1.46	0.309	0.725	0.758	1.74	2.70	1.53	2.01	2.87	3.08	2.66	1.78	0.442	10.7	3.06	3.22	0.528	8.66	3.33	0.000
C17	1.50	0.322	0.522	0.575	1.06	1.70	0.739	0.896	1.64	1.90	1.39	1.46	0.347	5.57	1.70	2.13	0.386	5.02	1.33	0.000
C18	1.11	0.311	0.554	0.541	2.02	2.46	0.938	1.18	1.72	1.98	1.74	1.39	0.439	9.11	2.57	2.84	0.437	7.44	3.44	0.000
C19	0.574	0.235	0.32	0.413	0.387	0.725	0.449	0.867	0.860	1.09	0.834	0.677	0.193	4.11	1.62	1.02	0.172	3.15	1.66	0.000
C20	0.698	0.317	0.412	0.367	1.09	1.55	1.13	1.20	1.60	2.09	1.83	1.22	0.225	7.80	2.83	2.09	0.171	5.66	2.54	0.000
C21	0.319	-	-	-	0.508	0.591	0.381	0.468	0.848	0.943	0.629	0.465	0.080	2.65	0.746	0.791	0.101	2.70	1.44	0.000
C22	0.507	0.202	0.403	0.259	0.988	1.55	0.940	0.912	1.50	1.89	1.57	1.01	0.156	6.06	1.90	1.55	1.27	5.29	2.63	0.000
C23	0.406	0.151	0.275	0.218	0.968	0.804	0.663	0.717	1.26	1.23	1.15	0.709	0.107	5.44	2.47	1.27	0.054	3.46	1.83	0.000
C24	0.977	0.332	0.723	0.522	1.40	2.15	1.48	1.22	2.33	2.57	2.47	1.50	0.258	7.90	2.55	2.24	0.213	6.75	3.08	0.000
C25	0.565	0.315	0.695	0.480	0.828	1.31	0.763	0.913	1.51	1.34	1.20	0.903	0.236	5.66	1.51	1.45	0.186	3.95	1.87	0.000
C26	0.933	0.215	0.469	0.287	1.65	2.76	1.29	1.47	2.86	2.47	2.19	1.40	0.192	7.95	2.81	2.01	0.314	6.59	2.49	0.000
C27	0.566	0.211	0.461	0.322	1.45	1.48	0.912	0.804	1.68	1.60	1.26	1.03	0.256	6.88	2.46	1.68	0.201	4.40	1.82	0.000
C28	0.772	0.138	0.720	0.211	1.07	1.82	1.03	1.03	1.70	2.11	1.85	1.07	0.201	6.61	2.64	1.70	0.185	4.69	1.86	0.000
C29	0.575	0.223	0.482	0.377	1.01	1.03	0.818	0.951	1.33	1.32	1.17	0.862	0.221	6.48	2.28	1.54	0.131	4.02	1.44	0.000
C30	0.904	0.106	0.491	0.179	1.44	2.25	1.22	1.27	1.88	2.28	1.71	1.21	0.190	9.24	3.43	2.08	0.404	5.10	1.88	0.000
C31	0.747	0.267	0.496	0.524	0.945	1.55	1.26	0.860	1.89	1.70	1.28	1.27	0.477	8.99	3.83	2.24	0.897	4.41	1.65	0.000
C32	0.497	0.128	0.361	0.176	0.816	1.450	0.660	0.787	1.25	1.41	1.43	0.714	0.200	4.21	1.03	1.12	0.042	3.48	1.24	0.000
C33	0.225	-	-	-	0.321	0.448	0.286	0.283	0.560	0.497	0.416	0.267	0.090	2.97	0.19	0.441	0.383	1.50	0.727	0.000
C34	0.301	0.091	0.446	0.160	0.529	0.842	0.428	0.395	0.924	1.12	0.990	0.590	0.171	3.42	1.03	2.09	2.16	2.22	0.630	0.000
C35	0.160	0.111	0.26	0.303	0.427	0.580	0.407	0.290	0.604	0.626	0.430	0.411	0.233	1.99	0.56	1.49	1.39	1.57	0.503	0.000
C36	0.172	0.101	-	0.141	0.177	0.906	0.301	0.253	0.553	0.660	0.622	0.328	0.183	2.28	0.55	0.728	0.292	1.83	1.090	0.000
C37	0.002	-	-	-	-	0.01	-	-	0.006	-	-	0.000	0.000	0.00	0.00	0.00	0.00	0.00	0.00	0.000
C38	0.090	0.171	-	0.191	-	0.56	0.459	-	0.701	0.606	0.618	0.345	0.453	0.00	0.00	0.134	0.233	1.610	0.699	0.000
Total	27.5	12.5	24.0	20.1	43.3	57.5	41.9	43.5	58.3	79.4	70.5	43.3	8.91	242	66.1	65.92	3.15	209	74.6	0.002

Supplementary Table S2 Compound-specific stable carbon isotope ratio of individual linear alkanes of the Dicrateria strains grown with different liquid media or different conditions.

Strain	Arctic strain*	NIES1001*	NBRC102791 *	RCC4214*	RCC4578*	RCC5635*	RCC5639*	Arctic strain		Arctic strain		Arctic strain		Arctic strain	
Growth medium	f/2	ESM	K	K/2	K/2	K	K	f/2		f/2		f/2		f/2	
Growth condition	Continuous light at 20 °C	Continuous light at 20 °C	Continuous light at 20 °C	Continuous light at 20 °C	Continuous light at 20°C	Continuous light at 20 °C	Continuous light at 20 °C	light (24 h) 20 °C		dark (24h) 20 °C		light (24 h) under low temperature (4 °C)		light (24 h) with nitrogen deficiency 20 °C	
	‰	‰	‰	‰	‰	‰	‰	‰	Standard deviation (*n* = 6)	‰	Standard deviation (*n* = 3)	‰	Standard deviation (*n* = 3)	‰	Standard deviation (*n* = 3)
C10	-66.3	-67.3	-66.9	-65.9	-64.1	-64.6	-68.9	-66.5	2.22	-67.7	1.02	-67.2	0.421	-67.9	0.919
C11	-65.8	-62.9	-63.8	-63.0	-58.7	-61.5	-63.9	-65.1	2.94	-67.5	1.98	-67.4	1.14	-63.0	1.10
C12	-25.0	-24.7	-24.8	-21.4	-27.6	-21.9	-22.8	-22.4	1.00	-22.5	1.69	-22.9	2.55	-22.9	0.830
C13	-20.0	-23.6	-25.2	-24.1	-18.6	-17.3	-23.6	-21.4	2.40	-23.0	1.57	-22.9	2.61	-21.3	0.702
C14	-26.4	-24.6	-23.6	-19.1	-25.1	-22.4	-24.1	-25.0	1.51	-26.0	3.77	-23.5	1.93	-25.7	0.318
C15	-24.0	-26.4	-24.4	-23.4	-26.2	-25.1	-26.3	-27.9	1.74	-28.3	4.73	-26.9	0.437	-27.8	1.87
C16	-27.8	-24.7	-28.5	-21.1	-22.9	-24.0	-24.6	-22.7	2.10	-23.6	3.19	-24.4	0.614	-24.1	1.82
C17	-26.0	-23.9	-25.4	-30.0	-23.8	-24.1	-26.2	-26.0	3.02	-27.9	7.60	-27.2	1.41	-27.3	2.49
C18	-27.5	-16.4	-21.2	-17.8	-20.0	-21.0	-18.6	-21.7	1.47	-22.5	1.71	-23.5	3.52	-21.8	3.34
C19	-30.7	-17.0	-25.4	-23.3	-20.5	-21.2	-22.6	-26.4	1.44	-25.7	3.18	-25.4	1.74	-25.9	3.55
C20	-30.2	-27.9	-27.0	-27.2	-27.3	-21.9	-21.4	-23.1	1.32	-25.2	2.40	-25.4	2.21	-27.4	0.880
C21	-33.1	-14.6	-22.2	-30.4	-25.4	-27.9	-31.5	-26.9	2.90	-23.9	3.35	-23.9	3.14	-24.4	4.19
C22	-31.0	-28.6	-26.3	-25.3	-25.2	-27.8	-25.8	-27.3	1.15	-27.9	1.51	-22.9	4.49	-26.3	1.47
C23	-30.5	-21.8	-28.7	-28.8	-28.8	-29.1	-32.8	-26.4	1.13	-27.1	2.69	-26.1	1.31	-25.5	2.36
C24	-29.3	-27.1	-19.7	-25.9	-28.9	-27.9	-20.1	-27.5	1.58	-28.7	1.84	-27.0	2.53	-27.4	1.72
C25	-30.0	-28.5	-	-31.1	-26.5	-28.6	-29.9	-25.3	1.85	-28.5	3.22	-27.7	2.19	-27.7	1.05
C26	-29.7	-27.8	-33.6	-31.2	-29.0	-30.9	-26.2	-28.2	3.55	-26.1	0.698	-28.0	-	-25.9	1.10
C27	-26.6	-33.7	-	-31.8	-31.1	-30.1	-40.7	-29.6	3.69	-33.2	5.63	-25.8	-	-27.1	4.44
C28	-28.5	-33.1	-	-	-32.1	-31.0	-39.9	-30.2	6.13	-33.7	2.38	-28.6	-	-33.6	0.928
C29	-28.9	-36.3	-45.2	-39.2	-33.6	-34.6	-38.8	-39.0	4.60	-27.1	3.91	-28.7	-	-31.9	5.05
C30	-28.4	-37.4	-	-45.7	-36.3	-39.2	-36.5	-40.0	3.46	-34.9	4.39	-36.8	-	-39.4	4.67
C31	-29.2	-	-	-	-	-	-	-29.3	-	-	-	-	-	-	-
C32	-29.2	-41.1	-24.0	-35.8		-27.1	-22.5	-33.7	9.8	-34.0	6.27	-38.0	-	-35.5	5.53
C33	-27.9	-33.7	-	-26.9	-38.0	-31.9	-48.1	-28.7	7.51	-35.5	8.87	-33.7	-	-30.9	2.99
C34	-27.6	-	-	-	-	-	-	-		-	-	-	-	-	-
C35	-29.0	-	-	-	-	-	-	-30.0	14.4	-37.8	-	-27.8	-	-42.9	7.41
C36	-28.5	-27.4	-	-	-	-	-19.0	-		-	-	-	-	-	-
C37	-31.6	-	-	-	-	-	-	-	-	-	-	-	-	-	-
C38	-34.8	-	-	-	-	-	-	-	-	-	-	-	-	-	-

"-" means "not detected".

* Experiment with no replication.
